# Beyond Mankind, Self-Illusion and Medical Expertise of Artificial Intelligence in the Era of COVID-19

**DOI:** 10.1093/ofid/ofag188

**Published:** 2026-04-02

**Authors:** Arthur Claessens, Yann-Erick Claessens, Aurélien Lambert, William Gehin

**Affiliations:** Department of Medical Oncology, Institut de Cancérologie de Lorraine, Vandoeuvre-lès-Nancy, Grand-Est, France; Department of Emergency Medicine, Princess Grace Hospital Center, Monaco, Monaco; Department of Medical Oncology, Institut de Cancérologie de Lorraine, Vandoeuvre-lès-Nancy, Grand-Est, France; Academic Department of Radiation Therapy & Brachytherapy, Institut de Cancérologie de Lorraine, Vandoeuvre-lès-Nancy, Grand-Est, France

To the editor—In our article “Self-Illusion and Medical Expertise in the Era of COVID-19” [1], published in Open Forum for Infectious Diseases, we demonstrated that during the spring 2020 COVID-19 pandemic, individuals—regardless of their scientific background exhibited marked overconfidence in their knowledge of SARS-CoV-2, despite widespread misinformation in the media. A survey of more than 2000 participants in the Principality of Monaco revealed a classic Dunning-Kruger effect: respondents expressed great certainty in their answers, but were often wrong, especially on controversial or sensational topics [2].

To assess whether large language models (LLMs) exhibit similar cognitive biases, we submitted the same nine questions to a panel of LLMs, asking them to quantify their level of confidence in each answer as if they were responding in the context of the spring 2020 pandemic in France. LLMs have shown very promising overall results with an accuracy rate of 81.4%. It is interesting to note that each of the LLMs had a high level of confidence (>7/10) in their answers, higher compared with human's historic cohort, regardless of whether they were accurate. If the sources cited in each answer came from online articles that complied with the time frame defined in the prompt, their interpretation seemed superficial, lacked nuance, and tended to extrapolate the most “catastrophic” or “spectacular” data. This was particularly evident in questions 8 and 9, where responses drew on a variety of sources without acknowledging temporal inconsistencies or mistaking simple hypotheses for facts. It can be observed that the self-confidence of human respondents was lower compared with LLMs in providing their responses (Table 1). The LLMs were queried in January 2026 using prompts that expressly asked them for responses as if they were addressing the context of the spring 2020 pandemic in France. Although their knowledge cutoff dates (2023) include information from after 2020, this approach aimed to simulate the knowledge framework of the time through constrained prompts. However, the influence of knowledge from after 2020 represents a possible methodological limitation, as it may introduce retrospective bias into their responses.

**Table 1.** Answers by LLMs of 2020 Claessens et al. Survey on COVID-19 Knowledge

LLM Assessed	Question	1	2	3	4	5	6	7	8	9	Overall
DeepSeek v3.1	Good Answer	Yes	Yes	Yes	Yes	Yes	Yes	Yes	No	No	78%
	Confidence (1 to 10)	8	8	7	6	9	8	9	8	7	7.78
	Mistake	-	-	-	-	-	-	-	Overestimate	Overestimate	…
Mistral Large 2	Good Answer	Yes	Yes	Yes	Yes	Yes	Yes	Yes	Yes	No	89%
	Confidence (1 to 10)	8	6	7	5	9	8	7	7	7	7.11
	Mistake	-	-	-	-	-	-	-	-	Overestimate	…
ChatGPT-5	Good Answer	Yes	Yes	Yes	Yes	Yes	Yes	Yes	No	No	78%
	Confidence (1 to 10)	8	7	7	6	10	9	10	8	7	8.00
	Mistake	-	-	-	-	-	-	-	Underestimate	Overestimate	…
Historical cohort from Claessens et al. [1]	Good Answer (percentage)	.31	.62	.65	.18	.27	.23	.46	.39	.31	35%
	Confidence (1 to 10) good responders	5.9	6.7	6.5	3.8	2.5	6.2	5.2	5.6	5.1	5.2
	Confidence (1 to 10) bad responders	6.0	5.7	4.7	2.5	2.4	4.1	4.4	5.1	4.2	4.3

LLM’s Self-estimated Confidence is Ranged from 1 (Minimal Confidence) to 10 (Maximal Confidence). Overall LLM’s Self-estimated Confidence is the Mean of 9 Answers for Each LLM. For reference, Results From the Claessens et al. Cohort [1] Associated with Mean Self-estimated Level of Confidence per Question.

These findings raise serious concerns about the role of generative AI in disseminating information and integrating it into all aspects of society. While our previous research highlighted the human's overconfidence bias in their knowledge about the pandemic [1], this exploratory study suggests that LLMs may replicate or even amplify these effects. The tendency to present unreliable or incomplete information with unjustified certainty underscores the need for critical evaluation of AI-generated content. Just as governments worldwide were urged to combat “infodemics” during the pandemic [2], users and developers must now address the risks of AI-generated overconfident responses, notably when LLMs lack contextual precision.

To address the overconfidence of LLMs, a three-layer audit approach is advisable, and can be tested on a randomized sample of the initial questions with an agent with a generative node and a control node (known as a ReACT protocol) [3]. First, a systematic reformulation of prompts could require a step-by-step rationale for each answer, strict temporal framing, and the requirement to cite dated sources. Second, the introduction of a second LLM to audit the responses of the first LLM could help to ensure that the responses are accurate and facts reliable, identifying temporal inconsistencies, unfounded extrapolations, or factual errors would allow for internal quality control, similar to peer review. Third, human supervision focused on a sample of responses (unrevised vs expert-reviewed) would assess whether this external validation improves the alignment between stated confidence and accuracy. This approach, inspired by cross-validation protocols, may enable LLMs to independently verify the absence of hallucinations and limit overconfidence bias.

This preliminary research identifies a Dunning-Kruger effect in LLMs’ self-assessment of confidence, where high confidence scores do not correspond to actual accuracy. This highlights a critical limitation: even when LLMs quantify their own confidence, this indicator does not guarantee the reliability of their results. Far from being reassuring, the confidence levels reported by LLMs can mislead users by making potentially incorrect or superficial responses appear to be reliable. These results reinforce the need to encourage critical thinking when using AI-generated content [4, 5].
